# Strongyloides stercoralis Hyperinfection Syndrome in Pregnancy

**DOI:** 10.7759/cureus.43568

**Published:** 2023-08-16

**Authors:** Allison N Akers, Adriana Vest, Claudio V Schenone, Alejandro Rodriguez

**Affiliations:** 1 Obstetrics and Gynecology, University of South Florida, Tampa, USA

**Keywords:** immunosuppressed, strongyloidiasis, placental abruption, antenatal corticosteroid use, parasitic infections in pregnancy, hyperinfection syndrome, strongyloides stercoralis

## Abstract

Strongyloidiasis is a parasitic infection with a high global burden of disease. Hyperinfection syndrome is a life-threatening complication that predominantly affects immunosuppressed individuals, such as those receiving corticosteroid treatment. Despite its worldwide prevalence, little is known about the clinical effects of this condition on the feto-maternal dyad during pregnancy. We present a case of placental abruption leading to preterm delivery in a pregnancy complicated by *Strongyloides stercoralis* hyperinfection syndrome following antenatal corticosteroid use. Although rare, this condition is associated with high mortality rates and adverse pregnancy outcomes. Therefore, screening at-risk individuals may be warranted in pregnancies where antenatal corticosteroid administration is considered.

## Introduction

*Strongyloides stercoralis* is a human intestinal parasite responsible for a spectrum of clinical syndromes ranging from asymptomatic to disseminated disease [1]. Although distributed worldwide, strongyloidiasis cases are most frequently seen in tropical and subtropical regions or populations with low socioeconomic status [2]. Despite the global prevalence of parasitic infections in pregnancy, maternal and fetal outcomes of* S. stercoralis* infection in pregnancy are poorly documented [3]. We present an unusual case of placental abruption leading to preterm delivery in the setting of *Strongyloides stercoralis* hyperinfection syndrome during pregnancy.

## Case presentation

**Figure 1:**
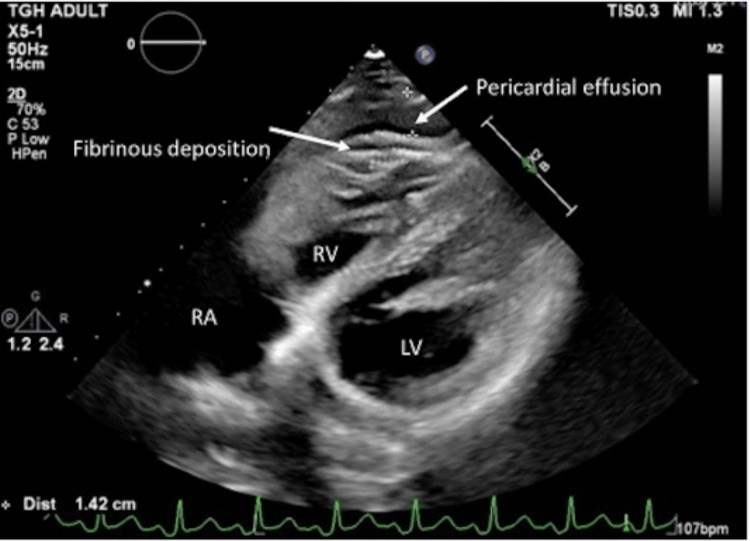
Transthoracic echocardiogram demonstrating small to moderate anterior pericardial effusion with associated fibrinous deposition

A 23-year-old gravida 5, para 0, Honduran female at 33 weeks presented with vomiting and a syncopal episode. During admission, an esophagogastroduodenoscopy (EGD) was performed and diagnostic for *Helicobacter pylori* gastritis. Given the potential for preterm delivery, the patient received a course of antenatal corticosteroid therapy for fetal maturation prior to the procedure. She was started on pantoprazole and famotidine and was discharged home in stable condition. Four days later, reports of ongoing nausea and vomiting, syncopal episodes, loss of consciousness at home, and tachycardia on evaluation prompted readmission. Initial evaluation was pertinent for mildly elevated troponins and maternal echocardiogram showing moderate pericardial effusion with layering echodensities suggestive of fibrinous exudates (Figure 1).

A decision was made in consultation with cardiology to ensure adequate hydration and repeat echocardiogram in six weeks.

On hospital day three, the patient reported vaginal bleeding and abdominal pain. An evaluation revealed progressive cervical dilation, blood in the vaginal vault, and non-reassuring fetal testing. An emergent cesarean delivery was recommended in the setting of suspected placental abruption, preterm labor, and persistent non-reassuring fetal tracing. Intraoperatively, the patient received dexamethasone for nausea. The delivery was uncomplicated and placental abruption was confirmed upon specimen examination. The infant weighed 2230 grams at delivery with Apgars 4 and 8 at one and five minutes of life, respectively. The infant was admitted to the Neonatal Intensive Care Unit (NICU) for 17 days.

**Figure 2:**
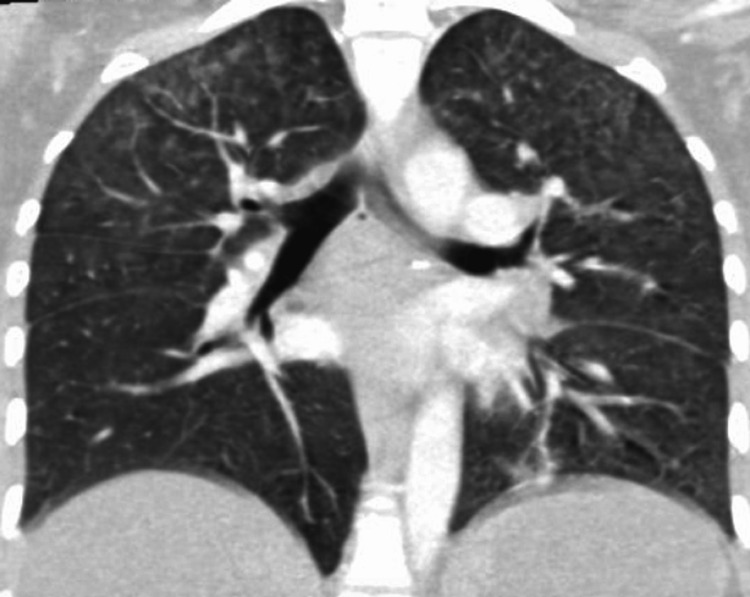
CT pulmonary angiogram (CTPA) showing ground glass nodules suggestive of hypersensitivity pneumonitis

On postoperative day one, the patient's liver enzymes were elevated. A complete metabolic panel, complete blood count with differential, hepatitis panel, and liver ultrasound (US) examination were unremarkable. A day later, recurrent tachycardia and inability to tolerate diet due to emesis prompted further evaluation. A CT pulmonary angiogram (CTPA) revealed ground glass nodules suspicious for hypersensitivity pneumonitis but was otherwise negative for pulmonary embolism (Figure 2).

**Figure 3:**
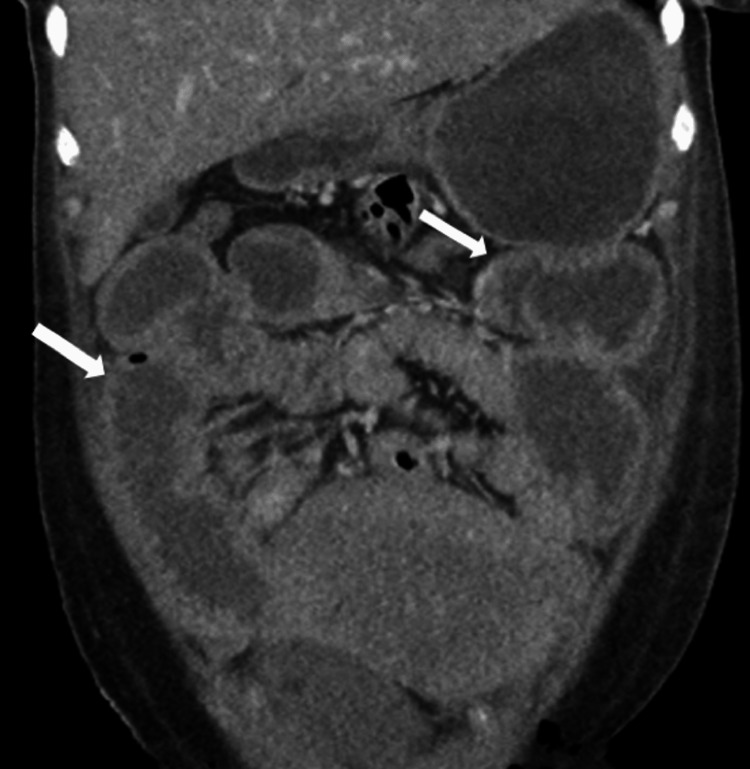
CT abdomen and pelvis also showed thickening of the small bowel and cecum, suggestive of a superimposed infectious or inflammatory enterocolitis

Additional findings included hypoactive bowel sounds, abdominal distension, and suspicion of small bowel obstruction vs. postoperative ileus on abdominal X-ray. Furthermore, a CT abdomen and pelvis showed thickening of the small bowel and cecum, suggesting a superimposed infectious or inflammatory enterocolitis (Figure 3).

Given no obvious etiology of small bowel obstruction, she was managed conservatively in consultation with a multidisciplinary team.

The patient’s clinical course failed to improve despite conservative measures, and she was diagnosed with sepsis on postoperative day 8 in the setting of fever, tachycardia, tachypnea, leukocytosis, and lactic acidosis. Cultures were obtained, intravenous antibiotics were initiated, and she was transferred to the Intensive Care Unit (ICU). Blood cultures grew coagulase-negative *Staphylococcus​​​​​​​ aureus* and *Escherichia coli*, stool ova and parasites and sputum cytology were positive for *S. stercoralis*. In consultation with infectious disease specialists, a hyperinfection syndrome diagnosis was made. The patient’s clinical course improved significantly following treatment with ivermectin and meropenem, and she was discharged home on postoperative day 43.

## Discussion

Strongyloidiasis is a parasitic infection with an estimated seroprevalence of up to 12.2% in migrants from endemic areas [2]. The cycle begins with rhabditiform larvae penetrating the human skin. After entering venous circulation, the larvae travel to the lungs and ascend the tracheobronchial tree and into the gastrointestinal tract, where the adult female reproduces [1,4]. While most cases of *S. stercoralis* are asymptomatic, severe cases have been described. Specifically, hyperinfection syndrome complicates 2.5% of cases and is characterized by massive larval invasion of the lungs and other tissues. Mortality rates associated with this condition are as high as 60-85% in immunocompromised hosts [4-5]. There is ample evidence linking parasitic infections in pregnancy to anemia, malnutrition, fetal growth restriction (FGR), preterm birth, and low birth weight [6]. In contrast, the literature specific to *S. stercoralis* hyperinfection syndrome in pregnancy is limited to case reports [3]. In a prospective Guatemalan study, high levels of helminthic infection were associated with an increased risk of FGR. However, this same association was not seen in *S. stercoralis* infection [6]. Buresch et al. described an intrauterine fetal demise associated with *S. stercoralis* hyperinfection syndrome, though the specific cause of fetal death was not specified [3]. To our knowledge, this represents the first case of placental abruption in the setting of *S. stercoralis* hyperinfection. Intrauterine infection is a well-known risk factor for this complication [7]. However, the role of extrauterine infections as a precipitating factor for placental abruption is less clear. In our case, the absence of traditional risk factors and the temporal relationship with *S. stercoralis* hyperinfection syndrome points to this condition as a plausible culprit.

Previous authors have exposed the association between treatment with glucocorticoids and the onset of hyperinfection syndrome in non-pregnant adults [1,8]. Antenatal steroid administration has also been implicated [3,5]. Based on available data, the known benefits of antenatal corticosteroids likely outweigh the risks of precipitating *S. stercoralis* hyperinfection syndrome, an uncommon complication. The COVID-19 pandemic presented a similar dilemma with the increased use of dexamethasone in hospitalized patients with this condition. Some have proposed screening and presumptive treatment with ivermectin for COVID-19 patients at high risk of *S. stercoralis *infection who are candidates for dexamethasone [8]. In pregnancy, similar suggestions have been made for at-risk patients receiving antenatal corticosteroids [2]. Although limited, safety reports on human pregnancies exposed to ivermectin do not suggest adverse outcomes [2,9]. Given the high mortality rates of *S. stercoralis* hyperinfection syndrome, the benefits of screening and treatment of at-risk patients receiving antenatal corticosteroids may outweigh the risks of ivermectin use in this population. The 2016 Committee to Advise on Tropical Medicine and Travel for Canada provided stratified risk categories, which may be helpful [5,10].

## Conclusions

In summary, strongyloidiasis is a common parasitic infection that may be fatal when complicated by hyperinfection syndrome. Although limited, evidence suggests this condition may be associated with adverse obstetric outcomes, highlighting the need to document maternal and fetal outcomes of *strongyloidiasis *during pregnancy and to keep *S. stercoralis* hyperinfection syndrome on the differential in at-risk patients with suggestive symptoms who have received antenatal corticosteroids.
